# Mutation Analysis of the Common Deafness Genes in Patients with Nonsyndromic Hearing Loss in Linyi by SNPscan Assay

**DOI:** 10.1155/2016/1302914

**Published:** 2016-05-09

**Authors:** Fengguo Zhang, Yun Xiao, Lei Xu, Xue Zhang, Guodong Zhang, Jianfeng Li, Huaiqing Lv, Xiaohui Bai, Haibo Wang

**Affiliations:** ^1^Department of Otorhinolaryngology Head and Neck Surgery, Shandong Provincial Hospital Affiliated to Shandong University, Jinan 250022, China; ^2^Shandong Provincial Key Laboratory of Otology, Jinan 250022, China; ^3^Department of Otorhinolaryngology Head and Neck Surgery, People's Hospital of Linyi, Linyi, China

## Abstract

Hearing loss is a common sensory disorder, and at least 50% of cases are due to a genetic etiology. Although hundreds of genes have been reported to be associated with nonsyndromic hearing loss,* GJB2*,* SLC26A4*, and* mtDNA12SrRNA* are the major contributors. However, the mutation spectrum of these common deafness genes varies among different ethnic groups. The present work summarized mutations in these three genes and their prevalence in 339 patients with nonsyndromic hearing loss at three different special education schools and one children's hospital in Linyi, China. A new multiplex genetic screening system “SNPscan assay” was employed to detect a total of 115 mutations of the above three genes. Finally, 48.67% of the patients were identified with hereditary hearing loss caused by mutations in* GJB2*,* SLC26A4*, and* mtDNA12SrRNA*. The carrying rate of mutations in the three genes was 37.76%, 19.75%, and 4.72%, respectively. This mutation profile in our study is distinct from other parts of China, with high mutation rate of* GJB2* suggesting a unique mutation spectrum in this area.

## 1. Introduction

Hearing loss or deafness is one of the most common neurosensory disorders in humans affecting about 1 to 3 in 1000 children worldwide [1]. It has been estimated that 30,000 newborns are detected with congenital hearing loss per 20 million live births every year in China [2], and approximately 50% of hearing loss is caused by a genetic etiology. At least 70% of all cases are classified as nonsyndromic hearing loss (NSHL) manifesting with isolated hearing loss without other associated clinical features. Of all NSHL, 75%–80% are autosomal recessive hereditary hearing loss, 15%–20% are autosomal dominant, 5% are X-linked, and 1% are inherited by mitochondrial genes [3].

Many previous genetic screening studies have shown that a large proportion of NSHL is caused by the mutations of a few genes, such as gap junction beta-2 protein (*GJB2*), solute carrier family 26 member 4 (*SLC26A4*), and* mitochondrial DNA* (*mtDNA*)* 12SrRNA* [4, 5]. Therefore, mutation screening of these common deafness-causing genes is of vital importance during genetic testing and counseling of deafness.

China harboring the highest population in the world is consisting of 56 nationalities. The mutation spectrum of the common deafness genes may vary among different ethnic nationalities due to geographical and language separation. The city Linyi with the biggest population and the largest area in Shandong Province locates in the east part of China. Almost all the populations in this area are Han nationality. People in Linyi are always impressed by introversion and rusticity. Because of its desolate geographical condition, Linyi city is a relatively conservative area with a less migration of population and an obstructive communication with other regions. Also, it has a less-developed economics for a long time. But to our knowledge, no systematic mutation analysis of deaf patients in Linyi has been reported previously. Therefore, there is a reason to believe that mutation spectrum of common deafness genes in patients with NSHL in this area must have a unique and special characteristic.

Up to now, many screening methods are used to detect the mutations of hearing loss, for example, direct sequencing, microarray, and PCR-restriction fragment length polymorphism (PCR-RFLP). These methods are either too expensive or time-consuming. Du et al. established a universal genetic screening system of hearing loss called the SNPscan assay which could detect 115 mutations of* GJB2*,* SLC26A4*, and* mtDNA12SrRNA* genes [6]. These selected mutations accounted for up to 90% of hearing loss caused by the most common deafness-causing genes in Chinese population.

In this study, we performed a comprehensive analysis of 3 common deafness-causing genes,* GJB2*,* SLC26A4*, and* mtDNA12SrRNA*, by SNPscan assay in 339 patients with NSHL from Linyi in eastern China. The aim was to investigate the molecular etiology in order to provide effective genetic counseling for hearing loss patients in this area.

## 2. Materials and Methods

### 2.1. Recruitment of the Subjects

A total of 339 subjects with NSHL from unrelated families were included in this study. They came from three different special education schools and one children's hospital in Linyi. Clinical questionnaire and informed consent were obtained from all subjects or their parents on their behalf. Clinical questionnaire showed basic information, including name, age, family history, health condition of the mother during pregnancy, and a clinical record of the patient, such as infections, possible head or brain injury, and the use of aminoglycoside antibiotics [7]. Physical and neurological examination was performed with special attention to renal, electrocardiac, and ophthalmologic differences to exclude those with syndromic hearing loss. An audiologic evaluation was performed including otoscope examination, tympanometry, and pure-tone audiometry (PTA). Auditory brainstem response (ABR) was employed in subjects when they were too young to accomplish the PTA test, and temporal bone computed tomography (CT) scan was also performed on participants for diagnosis of enlarged vestibular aqueduct (EVA) or inner ear malformation.

The onset of hearing impairment was categorized as prelingual/early (≤6 years) and late (>6 years). The degree of hearing impairment was calculated on the average of PTA in the speech frequencies 0.25, 0.5, 1, 2, and 4 kHz. Normal hearing was classified as PTA ≤ 25 dB nHL, mild hearing loss as PTA > 25 dB nHL and ≤40 dB nHL, moderate hearing loss as PTA > 40 dB nHL and ≤60 dB nHL, severe hearing loss as PTA > 60 dB nHL and ≤80 dB nHL, and profound hearing loss as PTA > 80 dB nHL. To enroll in this study, all subjects should have bilateral, permanent sensorineural hearing impairment. This study was approved by the Ethics Committee of Shandong Provincial Hospital.

### 2.2. DNA Samples

Genomic DNA for all 339 patients was prepared from 2 mL of peripheral blood with AxyPrep Genomic Blood DNA Extraction Kit (AXYGEN, USA). For the evaluation of the quantity and quality of extracted DNA, spectrophotometry (UNICO 2100, USA) and 1% agarose gel electrophoresis were carried out according to routine methods [8].

### 2.3. SNPscan for Mutation Detection

Two customized multiplex SNPscan assays from Genesky Biotechnologies Inc. (Shanghai, China) were designed to capture a total of 115 mutations of the three common deafness-causing genes as previously described (Table S1 in Supplementary Material available online at http://dx.doi.org/10.1155/2016/1302914): one is 53-plex and the other is 62-plex [6]. As a high-throughput and cost-saving SNP genotyping method, the SNPscan assay is based on double ligation and multiplex fluorescence PCR as described [9–11]. The SNPscan assays were done according to the detailed protocol described by Du et al. [6].

### 2.4. Statistical Analysis

The statistical analysis was performed using SAS 9.1.3 software (SAS, USA).

## 3. Result

### 3.1. Characterization of the Deaf Probands

A total of 339 probands with NSHL were recruited in this sequencing study, including 304 simplex and 35 multiplex probands. Simplex probands refer to the sporadic patients whose families have no one else suffering from hearing loss. Multiplex probands mean that there is at least one first- or second-degree deaf relative in the family. Clinical characterization of the deaf probands was summarized in Table 1. The patient cohort consisted of 196 males and 143 females, and they were all Han Chinese. The ages of the probands varied between a few months and 24 years (mean 8.9 years, 95% CI 8.3–9.5 years). Only one proband had late-onset, progressive, and moderate sensorineural hearing loss. All other probands had prelingual or early-onset sensorineural hearing loss. Hearing tests demonstrated that the level of hearing loss was severe to profound in 328 patients. The remaining 11 patients showed moderate hearing loss.

**Figure 1:**
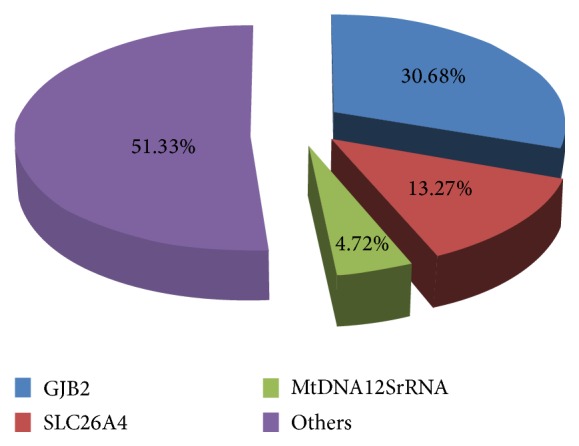
Distribution of the common deafness-causing genes in 339 NSHL patients.

Among all these patients, 48.67% were identified with hereditary hearing loss by means of the SNPscan assay (Figure 1). There were 104 patients (30.68%) affected by* GJB2* mutations: 49 homozygotes and 55 compound heterozygotes. 45 (13.27%) patients carried two* SLC26A4* pathogenic mutations: fourteen homozygotes and 31 compound heterozygotes. 16 (4.72%) patients harbored* mtDNA12SrRNA* mutations consisting of 15 homoplasmic mutations and 1 heteroplasmic mutation.

### 3.2. Mutations in* GJB2* Gene

Eleven variants were identified in this cohort. They were three frameshift deletions (c.176_191del16, c.235delC, and c.299_300delAT), two frameshift insertions (c.34_35insG and c.511_512insAACG), four missense mutations [c.109G>A (p.V37I), c.257C>G (p.T86R), c.427C>T (p.R143W), and c.571T>C (p.F191L)], one nonsense mutation c.9G>A (p.W3X), and one splicing site mutation IVS1+1G>A (shown in Table 2). Nine of them were pathological mutations which have been determined previously. The category of two nucleotide changes was unknown (c.9G>A, c.571T>C). The mutant alleles of* GJB2* accounted for 34.37% (233/678) of the total alleles in all patients (Table 3). The most common mutation allele of* GJB2* in this area was c.235delC with a mutant frequency of 20.65% (140/678). The second common was c.299_300delAT of 5.60% (38/678), followed by c.109G>A of 2.51% (17/678), c.34_35insG of 1.77% (12/678), c.176_191del16 of 1.33% (9/678), c.511_512insAACG of 1.18% (8/678), c.257C>G of 0.29% (2/678), c.427C>T of 0.29% (2/678), and IVS1+1G>A of 0.15% (1/678) (1/678) (Table 3).

A total of 104 patients (30.68%) were confirmed to be associated with hereditary hearing loss caused by* GJB2* mutation: 49 homozygotes (42 with the c.235delC allele, 4 with c.299_300delAT, and 3 with the c.109G>A allele) and 55 compound heterozygotes. Twenty patients (5.90%) had monoallelic variants in the heterozygous form: ten with c.235delC, eight with c.109G>A, one with c.299_300delAT, and one with c.511_512insAACG. Totally, 128 (37.76%, 128/339) patients had molecular defects in* GJB2* gene including three patients with c.571T>C mutation (Table 2).

The most common mutation allele of* GJB2* was c.235delC with a mutant frequency of 20.65% (140/678) affecting 98 patients. Among these patients carrying at least one c.235delC allele, there were 42 homozygotes, 46 compound heterozygotes, and 10 single heterozygotes.

### 3.3. Mutations in* SLC26A4* Gene

Nineteen variants were identified in this cohort, including 12 missense mutations [c.147C>G (p.S49R), c.269C>T (p.S90L), c.563T>C (p.I188T), c.589G>A (p.G197R), c.1173C>A (p.S391R), c.1174A>T (p.N392Y), c.1225C>T (p.R409C), c.1226G>A (p.R409H), c.1975G>C (p.V659L), c.1985G>A (p.C662Y), c.2027T>A (p.L676Q), and c.2168A>G (p.H723R)], four nonsense mutations [c.235C>T (p.R79X), c.946G>T (p.G316X), c.1318A>T (p.K440X), and c.1540C>T (p.Q514X)], two splicing site mutations (c.919-2A>G, c.1707+5 G>A), and one frameshift insertion mutation (c.1547_1548insC) (Table 5). Combining the prediction of SIFT and Polyphen-2 with the results of previous studies, all variants analyzed in our cohort were considered pathogenic mutations except c.147C>G (p.S49R) for an unknown significant pathogenicity (Table 4).

There were 45 (13.27%) patients who were confirmed to have inherited hearing loss because they carried two* SLC26A4* pathogenic mutations: fourteen homozygotes (13 c. IVS 7-2A>G and 1 c.2168A>G) and 31 compound heterozygotes. And 22 (6.49%) patients who were identified carried one* SLC26A4* pathogenic mutation. Thus, the detection rate of* SLC26A4* mutations was 19.76% (67/339) in this patient cohort. All patients carrying two mutations were diagnosed with EVA syndrome by CT scan of the temporal bone. And 17 out of 22 patients carrying one pathogenic mutation were diagnosed with EVA syndrome.

The most common mutation allele of* SLC26A4* was c.919-2A>G with a mutant frequency of 9.59% (65/678) affecting 52 patients. Among these patients carrying at least one c.919-2A>G mutation allele, there were 13 homozygotes, 27 compound heterozygotes, and 12 single heterozygotes. The second common mutation allele of* SLC26A4* was c.2168A>G with a mutant frequency of 2.21% (15/678).

### 3.4. Mutations in* mtDNA12SrRNA*

Sixteen patients (4.72%) carried an* mtDNA12SrRNA* mutation, where all of which were the m.1555A>G mutation containing 15 homoplasmic mutations and 1 heteroplasmic mutations. Ten of these patients had a clear history of aminoglycoside antibiotic use.

## 4. Discussion

Currently, many studies have reported that* GJB2*,* SLC26A4*, and* mtDNA12SrRNA* genes are the most common causes in Chinese NSHL population [12–14]. In the present study we have screened for the common deafness gene mutations of 339 NSHL children from Linyi, east part of China. SNPscan assay was used to perform genotyping detection of these three common deafness-causing genes. Compared with other methods, this technique is accurate, rapid, and economically effective. As the novel mutations have been reported successively, more mutation alleles should be added in the SNPscan assay.

### 4.1. *GJB2* Mutation Analysis

In this present study,* GJB2* mutations were detected in 37.76% (128/339) of all patients, including 30.68% (104/339) with two pathogenic mutations and 7.08% (24/339) with only one mutant allele. The c.235delC and c.299_300delAT were the most frequent mutations in the NSHL patients in Linyi, whereas the hotspot mutation in Xiamen, China, was c.109G>A [7].

The frameshift mutation c.235delC has been reported as the most common mutation causing premature protein termination in hearing impaired patients in East and Southeast Asia, while lower frequencies were reported in Europe and Oceania [15–24]. The c.235delC mutation allele frequency was 20.65% (140/678) in this work. In an earlier nationwide study, Dai et al. analyzed the* GJB2* mutation of 2063 unrelated NSHL students from 23 different regions of China, and the c.235delC mutation allele frequency was 12.34% (509/4,126) [25]. Compared with 12.34% in his study, the difference in the c.235delC mutation allele frequency was significant (*P* = 0.001), reflecting a certain distinction of the c.235delC mutation frequency in Linyi and other areas. There was a higher carrying rate of c.235delC in the Linyi deaf population.

### 4.2. *SLC26A4* Mutation Analysis

According to previous study, more than 200 mutations have been described in the* SLC26A4* gene with Pendred syndrome (PS) and enlarged vestibular aqueduct (EVA) syndrome (http://www.healthcare.uiowa.edu/labs/pendredandbor/) showing specific distinctions among racial backgrounds. Most of them are missense mutations, in addition to frameshift mutations, splice site mutations, insertions, or deletions [26].

The mutation hotspots of* SLC26A4* differed among different nations and areas. In the present study, the most common mutation in our patient cohort was c.IVS7-2A>G and the mutation allele frequency was 9.59% (65/678), whereas the mutation hot spots are p.T416P and c.IVS8+1G>A [27] in Northern Europe, p.H723R and c. IVS7-2A>G in South Korea [15], and p.H723R in Japan [28]. It was reported in a mutation study of a large cohort in Chinese deaf population that the most common* SLC26A4* mutation was also IVS7-2A>G (8.65%, 566/6,542) [12]. Although the mutation allele frequency was a little higher (9.59%) in our study, these frequencies of c.IVS7-2A>G are not significantly different (*P* = 0.41).

### 4.3. *mtDNA12SrRNA* Mutation Analysis

Patients with mutations in* mtDNA12SrRNA* can be affected with aminoglycoside antibiotic-induced deafness showing a hereditary model of maternal inheritance. And m.1494C>T and m.1555A>G are the most common mutations of this mitochondrial gene. Mutate rate of this gene varies among racial and geographic origins in populations with NSHL, with a frequency of 0.6–2.5% in Caucasians [29–32], 1.8% in Turks [30], 0.7% in Germans, 1.8% in Hungarians, and 2.4% in Poles [29]. In our study, we found that 4.72% of the patients were identified to have NSHL caused by the m.1555A>G mutation of* mtDNA12SrRNA* gene which was close to that observed by Dai et al. (3.87%) (*P* = 0.53).

In summary, we performed the first genetic analysis of hearing loss patients from Linyi, eastern part of China. According to genetic detections of* GJB2*,* SLC26A4*, and* mtDNA12SrRNA* via the SNPscan assay, the fact that almost half of the patients with nonsyndromic hearing loss appeared to have a genetic etiology shows that the SNPscan assay is an available diagnosis tool for studies on genetic hearing loss. The results of the detection suggest that* GJB2* mutations appear to be a major cause of congenital hearing loss in Linyi. The prevalence of* SLC26A4* mutations and* mtDNA12SrRNA* mutations was very close to other previous studies in Chinese deaf population. However, about 50% of NSHL patients are still not identified with a molecular etiology. For this reason, copy number variations (CNVs) of the three common deafness-causing genes need to be detected. Also, next generation sequencing (NGS) will be employed in screening other deafness-causing genes in the future.

## 5. Conclusions

In this study, we used the SNPscan assay technique to detect the 115 mutations of the three common deafness-causing genes in 339 nonsyndromic hearing loss patients from Linyi, China. And we found that the mutation profile was distinct from other parts of China suggesting a unique mutation spectrum in this area. Also, the SNPscan assay was an available diagnosis tool for studies on genetic hearing loss.

## Supplementary Material

All the 115 mutations of *GJB2*, *SLC26A4*, *mtDNA12SrRNA* detected in this study were summarized in the supplementary material.

## Figures and Tables

**Table 1:** Deaf patients (*n* = 339) categorized by their clinical characteristics.

	Simplex (*n* = 304)	Multiplex (*n* = 35)
Sex		
Male	179	17
Female	125	18
Age at the test		
0–6 years	153	13
6–18 years	122	21
18–35 years	29	1
Age of onset		
Early onset (≤6 years)	303	35
Late onset (>6 years)	1	0
Severity of hearing impairment		
Mild	0	0
Moderate	11	0
Severe	56	5
Profound	237	30

**Table 2:** *GJB2* genotypes of deaf patients from Linyi.

Allele 1			Allele 2			Number of patients
Nucleotide change	Consequence or amino acid change	Category	Nucleotide change	Consequence or amino acid change	Category	
IVS1+1G>A	Splicing site	Pathogenic	c.9G>A	p.W3X	Unknown	1
34_35insG	Frameshift	Pathogenic	109G>A	p.V37I	Pathogenic	1
34_35insG	Frameshift	Pathogenic	176_191del16	Frameshift	Pathogenic	1
34_35insG	Frameshift	Pathogenic	235delC	Frameshift	Pathogenic	8
34_35insG	Frameshift	Pathogenic	299_300delAT	Frameshift	Pathogenic	2
109G>A	p.V37I	Pathogenic	109G>A	p.V37I	Pathogenic	3
109G>A	p.V37I	Pathogenic	235delC	Frameshift	Pathogenic	1
109G>A	p.V37I	Pathogenic	299_300delAT	Frameshift	Pathogenic	1
109G>A	p.V37I	Pathogenic	—			8
176_191del16	Frameshift	Pathogenic	235delC	Frameshift	Pathogenic	8
235delC	Frameshift	Pathogenic	235delC	Frameshift	Pathogenic	42
235delC	Frameshift	Pathogenic	257C>G	p.T86R	Pathogenic	2
235delC	Frameshift	Pathogenic	299_300delAT	Frameshift	Pathogenic	22
235delC	Frameshift	Pathogenic	427C>T	p.R143W	Pathogenic	2
235delC	Frameshift	Pathogenic	511_512insAACG	Frameshift	Pathogenic	3
235delC	Frameshift	Pathogenic	—			10
299_300delAT	Frameshift	Pathogenic	299_300delAT	Frameshift	Pathogenic	4
299_300delAT	Frameshift	Pathogenic	511_512insAACG	Frameshift	Pathogenic	4
299_300delAT	Frameshift	Pathogenic	—			1
511_512insAACG	Frameshift	Pathogenic	—			1
571T>C	p.F191L	Unknown	—			3

**Table 3:** Allele frequencies of *GJB2* mutations in 339 deaf patients from Linyi.

Mutations	Consequence	Number of alleles	Allele frequency (%)
IVS1+1G>A	Splice site	1	0.15
c.34_35insG	Frameshift	12	1.77
c.109G>A	p.V37I	17	2.51
c.176_191del16	Frameshift	9	1.33
c.235delC	Frameshift	140	20.65
c.257C>G	p.T86R	2	0.29
c.299_300delAT	Frameshift	38	5.60
c.427C>T	p.R143W	2	0.29
c.511_512insAACG	Frameshift	8	1.18

**Table 4:** *SLC26A4* genotypes of deaf patients from Linyi.

Allele 1			Allele 2			Number of patients
Nucleotide change	Consequence or amino acid change	Category	Nucleotide change	Consequence or amino acid change	Category	
c.147C>G	p.S49R	Unknown	—			1
c.235C>T	p.R79X	Pathogenic	c.919-2A>G	Splice site	Pathogenic	1
c.235C>T	p.R79X	Pathogenic	—			1
c.269C>T	p.S90L	Pathogenic	—			1
c.563T>C	p.I188T	Pathogenic	c.919-2A>G	Splice site	Pathogenic	1
c.589G>A	p.G197R	Pathogenic	c.919-2A>G	Splice site	Pathogenic	1
c.919-2A>G	Splice site	Pathogenic	c.919-2A>G	Splice site	Pathogenic	13
c.919-2A>G	Splice site	Pathogenic	c.946G>T	p.G316X	Pathogenic	1
c.919-2A>G	Splice site	Pathogenic	c.1174A>T	p.N392Y	Pathogenic	2
c.919-2A>G	Splice site	Pathogenic	c.1226G>A	p.R409H	Pathogenic	3
c.919-2A>G	Splice site	Pathogenic	c.1318A>T	p.K440X	Pathogenic	1
c.919-2A>G	Splice site	Pathogenic	c.1540C>T	p.Q514X	Pathogenic	1
c.919-2A>G	Splice site	Pathogenic	c.1547_1548InsC	Frameshift	Pathogenic	1
c.919-2A>G	Splice site	Pathogenic	c.1707+5 G>A	Splice site	Pathogenic	3
c.919-2A>G	Splice site	Pathogenic	c.1975G>C	p.V659L	Pathogenic	3
c.919-2A>G	Splice site	Pathogenic	c.1985G>A	p.C662Y	Pathogenic	1
c.919-2A>G	Splice site	Pathogenic	c.2027T>A	p.L676Q	Pathogenic	2
c.919-2A>G	Splice site	Pathogenic	c.2168A>G	p.H723R	Pathogenic	6
c.919-2A>G	Splice site	Pathogenic	—			12
c.946G>T	p.G316X	Pathogenic	c.2168A>G	p.H723R	Pathogenic	1
c.1173C>A	p.S391R	Pathogenic	c.2168A>G	p.H723R	Pathogenic	1
c.1225C>T	p.R409C	Pathogenic	—			1
c.1226G>A	p.R409H	Pathogenic	c.1975G>C	p.V659L	Pathogenic	1
c.1226G>A	p.R409H	Pathogenic	c.2168A>G	p.H723R	Pathogenic	1
c.1226G>A	p.R409H	Pathogenic	—			1
c.1975G>C	p.V659L	Pathogenic	—			1
c.2168A>G	p.H723R	Pathogenic	c.2168A>G	p.H723R	Pathogenic	1
c.2168A>G	p.H723R	Pathogenic	—			4

**Table 5:** Allele frequencies of *SLC26A4* mutations in 339 deaf patients from Linyi.

Mutations	Consequence	Number of alleles	Allele frequency (%)
c.235C>T	p.R79X	2	0.29
c.269C>T	p.S90L	1	0.15
c.563T>C	p.I188T	1	0.15
c.589G>A	p.G197R	1	0.15
c.919-2A>G	Splice site	65	9.59
c.946G>T	p.G316X	2	0.29
c.1173C>A	p.S391R	1	0.15
c.1174A>T	p.N392Y	2	0.29
c.1225C>T	p.R409C	1	0.15
c.1226G>A	p.R409H	6	0.88
c.1318A>T	p.K440X	1	0.15
c.1540C>T	p.Q514X	1	0.15
c.1547_1548InsC	Frameshift	1	0.15
c.1707+5 G>A	Splicing site	3	0.44
c.1975G>C	p.V659L	5	0.74
c.1985G>A	p.C662Y	1	0.15
c.2027T>A	p.L676Q	2	0.29
c.2168A>G	p.H723R	15	2.21

## References

[1] Mehl A. L., Thomson V. (2002). The Colorado newborn hearing screening project, 1992–1999: on the threshold of effective population-based universal newborn hearing screening. *Pediatrics*.

[2] Dai P., Liu X., Han D. (2006). Extremely low penetrance of deafness associated with the mitochondrial 12S rRNA mutation in 16 Chinese families: implication for early detection and prevention of deafness. *Biochemical and Biophysical Research Communications*.

[3] Bozdogan S. T., Kuran G., Yüregir Ö. Ö. (2015). The prevalence of Gap Junction Protein Beta 2 (GJB2) mutations in non syndromic sensorineural hearing loss in çukurova region. *Journal of International Advanced Otology*.

[4] Yuan Y., You Y., Huang D. (2009). Comprehensive molecular etiology analysis of nonsyndromic hearing impairment from typical areas in China. *Journal of Translational Medicine*.

[5] Xin F., Yuan Y., Deng X. (2013). Genetic mutations in nonsyndromic deafness patients of Chinese minority and han ethnicities in Yunnan, China. *Journal of Translational Medicine*.

[6] Du W., Cheng J., Ding H., Jiang Z., Guo Y., Yuan H. (2014). A rapid method for simultaneous multi-gene mutation screening in children with nonsyndromic hearing loss. *Genomics*.

[7] Jiang Y., Huang S., Deng T. (2015). Mutation spectrum of common deafness-causing genes in patients with non-syndromic deafness in the Xiamen Area, China. *PLoS ONE*.

[8] Bai X., Lv H., Zhang F. (2014). Identification of a novel missense mutation in the *WFS1* gene as a cause of autosomal dominant nonsyndromic sensorineural hearing loss in all-frequencies. *American Journal of Medical Genetics Part A*.

[9] Chen X., Li S., Yang Y. (2012). Genome-wide association study validation identifies novel loci for atherosclerotic cardiovascular disease. *Journal of Thrombosis and Haemostasis*.

[10] Wei J., Zheng L., Liu S. (2013). MiR-196a2 rs11614913 T>C polymorphism and risk of esophageal cancer in a Chinese population. *Human Immunology*.

[11] Zheng L., Yin J., Wang L. (2013). Interleukin 1B rs16944 G>A polymorphism was associated with a decreased risk of esophageal cancer in a Chinese population. *Clinical Biochemistry*.

[12] Dai P., Li Q., Huang D. (2008). *SLC26A4* c.919-2A>G varies among Chinese ethnic groups as a cause of hearing loss. *Genetics in Medicine*.

[13] Duan S.-H., Zhu Y.-M., Wang Y.-L., Guo Y.-F. (2015). Common molecular etiology of nonsyndromic hearing loss in 484 patients of 3 ethnicities in northwest China. *Acta Oto-Laryngologica*.

[14] Fang Y., Gu M., Wang C., Suo F., Wang G., Xia Y. (2015). * GJB2* as well as *SLC26A4* gene mutations are prominent causes for congenital deafness. *Cell Biochemistry and Biophysics*.

[15] Tsukamoto K., Suzuki H., Harada D. (2003). Distribution and frequencies of PDS (SLC26A4) mutations in Pendred syndrome and nonsyndromic lossassociated with enlarged vestibular aqueduct: a unique spectrum of mutations in Japanese. *European Journal of Human Genetics*.

[16] Lai C.-C., Chiu C.-Y., Shiao A.-S. (2007). Analysis of the *SLC26A4* gene in patients with Pendred syndrome in Taiwan. *Metabolism: Clinical and Experimental*.

[17] Huang S., Han D., Yuan Y. (2011). Extremely discrepant mutation spectrum of *SLC26A4* between Chinese patients with isolated Mondini deformity and enlarged vestibular aqueduct. *Journal of Translational Medicine*.

[18] Okamoto Y., Mutai H., Nakano A. (2014). Subgroups of enlarged vestibular aqueduct in relation to *SLC26A4* mutations and hearing loss. *Laryngoscope*.

[19] Hu H., Wu L., Feng Y. (2007). Molecular analysis of hearing loss associated with enlarged vestibular aqueduct in the mainland Chinese: a unique *SLC26A4* mutation spectrum. *Journal of Human Genetics*.

[20] Yang J.-J., Tsai C.-C., Hsu H.-M., Shiao J.-Y., Su C.-C., Li S.-Y. (2005). Hearing loss associated with enlarged vestibular aqueduct and Mondini dysplasia is caused by splice-site mutation in the PDS gene. *Hearing Research*.

[21] Choi B. Y., Madeo A. C., King K. A. (2009). Segregation of enlarged vestibular aqueducts in families with non-diagnostic *SLC26A4* genotypes. *Journal of Medical Genetics*.

[22] Nishio S.-Y., Usami S.-I. (2015). Deafness gene variations in a 1120 nonsyndromic hearing loss cohort: molecular epidemiology and deafness mutation spectrum of patients in Japan. *The Annals of Otology Rhinology and Laryngology*.

[23] Wattanasirichaigoon D., Limwongse C., Jariengprasert C. (2004). High prevalence of V37I genetic variant in the *connexin-26* (*GJB2*) gene among non-syndromic hearing-impaired and control Thai individuals. *Clinical Genetics*.

[24] Han S.-H., Park H.-J., Kang E.-J. (2008). Carrier frequency of *GJB2* (connexin-26) mutations causing inherited deafness in the Korean population. *Journal of Human Genetics*.

[25] Dai P., Yu F., Han B. (2009). *GJB2* mutation spectrum in 2,063 Chinese patients with nonsyndromic hearing impairment. *Journal of Translational Medicine*.

[26] Dossena S., Bizhanova A., Nofziger C. (2011). Identification of allelic variants of pendrin *(SLC26A4*) with loss and gain of function. *Cellular Physiology and Biochemistry*.

[27] Campbell C., Cucci R. A., Prasad S. (2001). Pendred syndrome, DFNB4, and PDS/*SLC26A4* identification of eight novel mutations and possible genotype-phenotype correlations. *Human Mutation*.

[28] Park H.-J., Lee S.-J., Jin H.-S. (2005). Genetic basis of hearing loss associated with enlarged vestibular aqueducts in Koreans. *Clinical Genetics*.

[29] Kupka S., Tóth T., Wróbel M. (2002). Mutation A1555G in the 12S rRNA gene and its epidemiological importance in German, Hungarian, and Polish patients. *Human Mutation*.

[30] Tekin M., Duman T., Boğoçlu G. (2003). Frequency of *mtDNA* A1555G and A7445G mutations among children with prelingual deafness in Turkey. *European Journal of Pediatrics*.

[31] Li R., Greinwald J. H., Yang L., Choo D. I., Wenstrup R. J., Guan M. X. (2004). Molecular analysis of the mitochondrial 12S rRNA and tRNASer(UCN) genes in paediatric subjects with non-syndromic hearing loss. *Journal of Medical Genetics*.

[32] Jacobs H. T., Hutchin T. P., Käppi T. (2005). Mitochondrial DNA mutations in patients with postlingual, nonsyndromic hearing impairment. *European Journal of Human Genetics*.

