# Supplementary material for: Mutation Analysis of the Common Deafness Genes in Patients with Nonsyndromic Hearing Loss in Linyi by SNPscan Assay
Source: Biomed Res Int. 2016 May 9;2016:1302914. doi: 10.1155/2016/1302914 (PMC4876198; doi:10.1155/2016/1302914)
Supplement: Supplementary file 1 — All the 115 mutations of GJB2, SLC26A4, mtDNA12SrRNA detected in this study were summarized in the supplementary material. [file 1302914.f1.pdf]

Supplement material

Table S1. 115 mutations of the 3 deafness causing genes detected in the study

gene	allele	Category
GJB2	IVS1+1G>A	Pathogenic
	1A>G	Pathogenic
	9G>A	Unknown
	23C>T	Pathogenic
	35delG	Pathogenic
	34_35insG	Pathogenic
	95G>T	Pathogenic
	95G>A	Pathogenic
	109G>A	Pathogenic
	134G>A	Pathogenic
	139G>T	Pathogenic
	157T>A	Pathogenic
	164C>A	Pathogenic
	167delT	Pathogenic
	176_191del16	Pathogenic
	187G>T	Pathogenic
	230G>A	Pathogenic
	232G>A	Pathogenic
	235delC	Pathogenic
	257C>G	Pathogenic
	283G>A	Pathogenic
	287C>G	Pathogenic
	299_300delAT	Pathogenic
	313_326del14	Pathogenic
	358_360delGAG	Pathogenic
	382A>G	Pathogenic
	408C>A	Pathogenic
	416G>A	Pathogenic
	427C>T	Pathogenic
	439G>A	Pathogenic
	493C>T	Pathogenic
	511_512insAACG	Pathogenic
	571T>C	Unknown
	583A>G	Pathogenic
	598G>A	Pathogenic
	605ins46	Pathogenic
	109G>T	Pathogenic
	147C>G	Unknown
	170C>A	Pathogenic
	227C>T	Pathogenic
	230A>T	Pathogenic

	235C>T	Pathogenic
	249G>A	Pathogenic
	269C>T	Pathogenic
	279T>A	Pathogenic
	281C>T	Pathogenic
	387delC	Pathogenic
	398C>T	Pathogenic
	404A>G	Pathogenic
	414delT	Pathogenic
	421T>C	Pathogenic
	439A>G	Pathogenic
	563T>C	Pathogenic
	589G>A	Pathogenic
	665G>T	Pathogenic
	668T>C	Pathogenic
	754T>C	Pathogenic
	766 -2A>G	Pathogenic
	907G>C	Pathogenic
	916_917insG	Pathogenic
SLC26A4	919-2A>G(ivs7-2)	Pathogenic
	946G>T	Pathogenic
	1001+1G>A	Pathogenic
	1022delC	Pathogenic
	1079C>T	Pathogenic
	1105A>G	Pathogenic
	1160C>T	Pathogenic
	1173C>A	Pathogenic
	1174A>T	Pathogenic
	1225C>T	Pathogenic
	1226G>A	Pathogenic
	1229C>T	Pathogenic
	1238A>G	Pathogenic
	1240-1243GAGA>AAAG	Pathogenic
	1262A>C	Pathogenic
	1264G>A	Pathogenic
	1318A>T	Pathogenic
	1327G>C	Pathogenic
	1334T>G	Pathogenic
	1336C>T	Pathogenic
	1340delA	Pathogenic
	1343C>A	Pathogenic
	1343C>T	Pathogenic
	1371C>A	Pathogenic
	1489G>A	Pathogenic

	1517T>G	Pathogenic
	1520delT	Pathogenic
	1522A>G	Pathogenic
	1540C>T	Pathogenic
	1547_1548InsC	Pathogenic
	1586T>G	Pathogenic
	1594A>C	Pathogenic
	1595G>T	Pathogenic
	1614+9C>T	Pathogenic
	1615A>G	Pathogenic
	1673A>T	Pathogenic
	1686_1687insA	Pathogenic
	1699A>T	Pathogenic
	1707+1G>A	Pathogenic
	IVS15+5G>A (1707+5 G>A)	Pathogenic
	1829C>A	Pathogenic
	1927G>T	Pathogenic
	1949T>A	Pathogenic
	1975G>C	Pathogenic
	1985G>A	Pathogenic
	1991C>T	Pathogenic
	2014G>A	Pathogenic
	2027T>A	Pathogenic
	2054G>T	Pathogenic
	2086C>T	Pathogenic
	2162C>T	Pathogenic
	2167C>G	Pathogenic
	2168A>G	Pathogenic
mtDNA12SrRNA	1494 C>T	Pathogenic
	1555 A>G	Pathogenic
